# Electrophysiological Representation of Scratching CPG Activity in the Cerebellum

**DOI:** 10.1371/journal.pone.0109936

**Published:** 2014-10-28

**Authors:** Lourdes Martínez-Silva, Elias Manjarrez, Gabriel Gutiérrez-Ospina, Jorge N. Quevedo

**Affiliations:** 1 Departamento de Fisiología, Biofísica y Neurociencias CINVESTAV, México City, México; 2 Instituto de Fisiología, BUAP, Puebla, México; 3 Instituto de Investigaciones Biomédicas - UNAM, México City, México; Scientific Institute Foundation Santa Lucia, Italy

## Abstract

We analyzed the electrical activity of neuronal populations in the cerebellum and the lumbar spinal cord during fictive scratching in adult decerebrate cats before and after selective sections of the Spino-Reticulo Cerebellar Pathway (SRCP) and the Ventral-Spino Cerebellar Tract (VSCT). During fictive scratching, we found a conspicuous sinusoidal electrical activity, called Sinusoidal Cerebellar Potentials (SCPs), in the cerebellar vermis, which exhibited smaller amplitude in the paravermal and hemisphere cortices. There was also a significant spino-cerebellar coherence between these SCPs and the lumbar sinusoidal cord dorsum potentials (SCDPs). However, during spontaneous activity such spino-cerebellar coherence between spontaneous potentials recorded in the same regions decreased. We found that the section of the SRCP and the VSCT did not abolish the amplitude of the SCPs, suggesting that there are additional pathways conveying information from the spinal CPG to the cerebellum. This is the first evidence that the sinusoidal activity associated to the spinal CPG circuitry for scratching has a broad representation in the cerebellum beyond the sensory representation from hindlimbs previously described. Furthermore, the SCPs represent the global electrical activity of the spinal CPG for scratching in the cerebellar cortex.

## Introduction

Rhythmic motor tasks, like scratching, walking or swimming, are due to the rhythmic activity of spinal interneurons termed central pattern generators (CPG), that control the alternation between flexor and extensor motoneurons. While the identity of these interneurons remains incomplete, some efforts to understand their spatiotemporal activation have demonstrated that spinal field potentials recorded during CPG activity take the shape of sinusoidal waves (SCDPs; sinusoidal-like cord dorsum potentials). Such SCDPs propagate along the spinal cord in *in vivo* decerebrate cats during fictive scratching [1], [2].

Scratching is a simple rhythmic motor task in which one hindlimb moves rhythmically, 3–5 Hz [3], to eliminate an irritant stimulus on the skin while the other limbs keep almost inactive. In order to control such scratching movements, the cerebellum plays an important role by integrating the sensory feedback and motor programs to coordinate the ongoing motor task [4]. Several studies suggest that the cerebellum receives an efferent copy of the motor program generated by the spinal CPG *via* the Ventral-Spino Cerebellar Tract (VSCT) and the Spino-Reticulo Cerebellar Pathway (SRCP) [5], [6], [7], [8], [9], [10], [11], [12], [13]. Moreover, Arshavsky and colleagues [9], [10], [11] demonstrated that the VSCT and the SRCP tracts exhibit a step cycle-related firing increase during the flexion or extensor phase of scratching, respectively. Several studies, employing extracellular field potentials and single unitary recordings, reinforce the possibility of rhythmic modulation on the cerebellar activity by spinal networks during rhythmic motor tasks. There is a cyclic modulation of Purkinje cells [14], [15], [16], [17], [18], [19], [20], [21], Golgi cells [22], and deep cerebellar nuclei [23], [24], [25], [26]. However, these studies relied on unitary neuronal activity, and so far, there is no evidence about the electrophysiological activity of neuronal populations in the cerebellar cortex during the spinal CPG activity. We hypothesize that the rhythmic activity associated to the spinal CPG for scratching is also expressed in the cerebellar cortex as the SCPs, with a defined representation mainly in the cerebellar vermis, where the VSCT and the SRCP have their main projections. The first aim of our study was to explore the distribution of the SCPs along the cerebellar cortex to determine the region in which they exhibit the larger amplitude, and to analyze the coherence with the sinusoidal spinal potentials during fictive scratching. The second aim of our study was to examine the effects of disrupting the VSCT and the SRCP on the SCPs in the cerebellar cortex. To this end, we performed selective sections of the VSCT and the SRCP at the appropriate spinal levels. These results are relevant because we characterize, for the first time. a defined representation of the CPG for scratching in the cerebellar cortex.

## Methods

### Ethics Statement

All the procedures described here comply with the guidelines contained in the National Institutes of Health *Guide for the Care and Use of Laboratory Animals* (USA) and were approved by the Institutional Animal and Use Committees in the “Centro de Investigación y de Estudios Avanzados” and the “Benemérita Universidad Autónoma de Puebla” (Mexico).

### Surgical Procedures

Experiments were performed on 19 adult cats, of either sex, weighing 3–5 kg. For surgery, anesthesia was induced and maintained with isoflurane (1%) delivered in oxygen (99%). A surgical plane of anesthesia was verified by monitoring the blood pressure and examining the lack of withdrawal reflex and muscle tone. One jugular vein was cannulated to administer drugs and fluids, and blood pressure was monitored from carotid artery. A tracheostomy was performed for anesthesia maintenance, and for artificial respiration after the decerebration. At the beginning of the surgery, atropine (0.05 mg/kg s.c.) was given to control excessive salivation and bronchial secretions, and dexamethasone (2 mg/kg i.v.) to prevent edema. A solution of 5% glucose and bicarbonate solution was delivered intravenously throughout the experiment at a rate of 5 ml/h. Supplemental saline and a plasma substitute (Gelafundin, 274 mOsm/l) were infused as required to maintain blood pressure. A catheter was inserted through the urethra to measure the urinary output and to control the fluids balance along the experiment.

**Figure 1:**
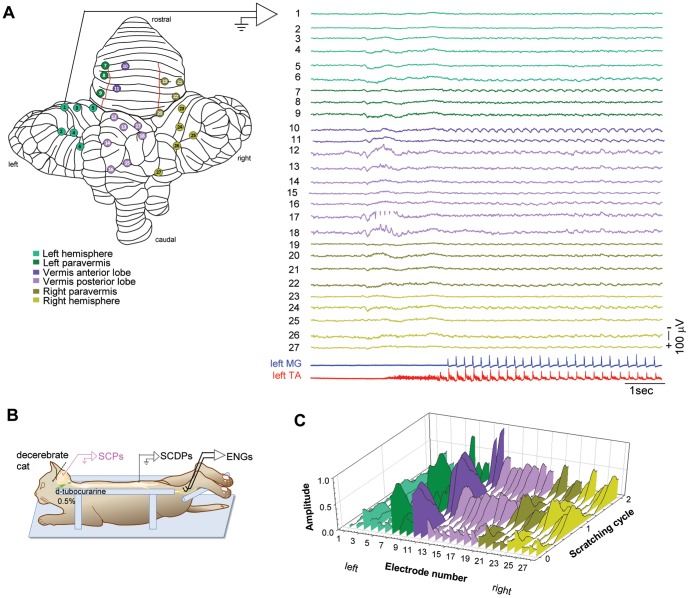
Sinusoidal cerebellar potentials (SCPS) during fictive scratching. ***A–B:*** Experiments are performed in adult decerebrate and paralyzed cats, with simultaneous recordings of the cerebellar, spinal and electroneurogram (ENG) activity, as illustrated in *B*. The left panel in *A* represents a dorsal view of an unfolded cerebellum of the cat showing 27 recording electrodes placed on several areas of the cortex surface to record the SCPs (see color nomenclature). The scratching activity is recorded from the flexor, tibialis anterior (TA, red), and the extensor, medial gastrocnemius (MG, blue) nerves (right panel, lower traces). Notice that the SCPs occur only during the phasic phase of scratching (flexor-extensor ENG alternation). ***C:*** Comparative distribution of the mean amplitude of the SCPs during two scratching cycles. The SCPs from the scratching episode in *A* are normalized, based on the flexor phase of the scratching cycle, and averaged. The highest amplitude of the SCPs is exhibited along the vermis (electrodes 10–18), mainly on the anterior lobe (see recordings 10 and 11 in *A*).

A bilateral dissection of the tibialis anterior (TA), the lateral gastrocnemius and soleus (LGS) and the medial grastrocnemius (MG) nerves was performed. Dissected nerves were placed on bipolar electrodes for stimulation and recording. The lumbosacral segments (L4-S1) of the spinal cord were exposed by laminectomy. In some experiments the low thoracic-lumbar (T13-L1) and the cervical (C4-C6) segments were also exposed, with the aim to selectively section the spino-cerebellar tracts. After these surgical procedures, the animal was transferred to a rigid stereotaxic frame. The vertebral column was fixed by means of spinal and pelvic clamps and the head immobilized by means of ear bars, eye pits and an upper jaw holder (see Figure 1B). Pools were made with the skin flaps around the exposed tissues, filled with mineral oil and warmed by radiant heat. The temperature was monitored by one thermode inserted into the esophagus and a second one placed in the back pool, and maintained at 37°C by a servo control system.

Following a craniotomy, a precollicular-postmammillary decerebration was performed with removal of both cerebral cortices and the tissues rostral to the transection. This rendered the animal totally insentient allowing the anesthesia to be discontinued. The animal was paralyzed with pancuronium bromide (0.6 mg/kg) and artificially ventilated. The end-tidal CO_2_ was kept at 3–3.5%. Finally, the cerebellar cortex was exposed and covered with gel mineral oil.

Animals were euthanized at the end of the experiment with an overdose of pentobarbital. Zero blood pressure indicated that the animal was euthanized.

### Electrophysiological recordings

Fictive scratching was evoked by tactile stimulation of left or right pinna, after topical application of d-tubocurarine (0.1–0.5%; Sigma) on the surface of C1–C2 cervical segments. Electroneurograms (ENGs) were recorded with bipolar hook silver electrodes placed in the distal end of the sectioned nerves (MG and TA). The cerebellar and spinal activity were recorded by means of silver ball electrodes, placed on the surface, with a reference electrode inserted into the neck and paravertebral muscles, respectively. In some experiments (n = 5), we recorded the neuronal electrical activity using an array of 30 electrodes (Ag-AgCl, 200 µm diameter) placed on the lumbosacral (L4-S1) surface of the spinal cord [1], and another one on the surface of the cerebellar cortex. The spontaneous activity, and the activity during scratching, were recorded monopolarly with a Synamps EEG amplifier (Neuroscan). The raw data were AC coupled, bandpass filtered (0.05–500 Hz) and sampled at a rate of 10 KHz. The cerebellar multi-electrode array allowed us to explore simultaneously both hemispheres and the vermal cortices, and to identify the regions that exhibited the SCPs of highest amplitude. We found that the vermal and paravermal regions exhibited the highest amplitude, and for this reason we focused on these regions. In five experiments, we used one silver ball electrode placed on the L6–L7 spinal border and one on the vermal lobule Vc. In nine experiments, we recorded cerebellar surface activity by means of one array of six electrodes placed on the vermis, and the spinal activity by means of one silver ball electrode placed on the L6-L7 spinal border. In these cases, we used custom-made AC-coupled amplifiers (Spinal Cord Research Center, SCRC; University of Manitoba, Canada). Raw data were collected by means of the Digidata 1440 card and the pClamp software v. 10.4 (Molecular Devices), with a bandpass of 0.3–10 KHz and a sampling rate of 10 KHz.

### Data analysis

#### Amplitude analysis of the SCPs

The amplitude (peak to peak) of the SCPs and SCDPs was measured from averaged and normalized cycles. Cycle normalization was based on the flexor ENG activity by using the Analysis Software of the Spinal Cord Research Centre of the University of Manitoba, Canada. Before normalizing and averaging the scratching cycles, the raw SCPs and SCDPs recordings were filtered (bandpass 1–20 Hz), and ENGs were filtered (bandpass 40–75 Hz) and rectified. The amplitude of the SCPs and SCDPs was usually normalized to the highest amplitude recorded for each scratching episode.

#### Frequency analysis of the SCPs and SCDPs

To extract the different frequency components of the SCPs and SCDPs, we computed the power spectrum density function (PSDF) based on the fast Fourier transform. The PSDF was calculated with the Clampfit software v.10.4 (Molecular devices) to find common peaks of frequency for the cerebellar, the spinal, and the ENG activity during scratching episodes, as well as during spontaneous activity. The PSDF plots show the power density, normalized to the maximum value versus the frequency, which was limited around the scratching frequency range (1–5 Hz).

Additionally, in order to compare the SCPs versus the SCDPs and the ENGs during scratching and at rest, we computed the cross-correlation *via* the mean coherence function (Cxy) using the Matlab software (The MathWorks Inc.). The value of the spino-cerebellar coherence function ranged from zero, indicating no correlation, to one, that indicates a perfect correlation, between X(f) and Y(f) at a given frequency, f.

In order to analyze variations of the spino-cerebellar correlation between the SCPs and the SCDPs in time and frequency domains, we computed the spino-cerebellar Wavelet Coherence Analysis (WCT) based on the short-time Fourier and the continuous wavelet transform functions [27], [28]. The spino-cerebellar WCT was performed offline on recorded data during scratching, including a few seconds before and after a scratching episode (spontaneous activity). To this end, we used the Matlab wavelet coherence package developed by Aslak Grinsted [29] and Torrence and Compo [27]. The WCT plots display regions with high spino-cerebellar coherence (dark red color indicates a perfect correlation; value = 1) in time (X-axis, ms) and frequency (Y-axis, Hz) domains. The color map outside the white shadow areas is statistically significant, according to the Monte Carlo estimation method [28]. The phase relationship between both time signals, X(t) and Y(t), is shown as black arrows pointing right or left, which indicates in-phase or anti-phase, respectively.

### Disruptions of the spinocerebellar tracts

The lateral funiculus, contralateral to the stimulated side to evoke fictive scratching, was sectioned between the T13 thoracic and L1 lumbar segments to disrupt the VSCT, by using fine scissors and forceps. The lateral funiculus, ipsilateral to the stimulated side to evoke fictive scratching, was sectioned at the cervical segment C5 to disrupt the SRCP [9], [11], [30]. After each section, the animal rested for 10 min to recover from the spinal shock, meanwhile the scratching CPG activity was recorded to verify that the descending scratching pathway was not disrupted. VSCT was sectioned first in 3/7, SRCP was sectioned first in 4/7, both VSCT and SRCP in 5/7, and dorsal columns in 3/7 experiments. At the end of these experiments the spinal cord was recovered for histological analysis.

### Histological evaluation of the lesions

In order to determine the extent of the spinal lesions, the spinal cord was removed and embedded in 10% formaldehyde during 24 hrs for fixing. After fixation, the spinal cord was immersed in methyl salicylate for one-two weeks in order to clear the tissue. Once the spinal cord looked transparent it was cut in serial transverse sections and placed on a glass slide. The slides were photographed with a digital camera (Canon S5IS) under the 10× objective of an upright microscope (Olympus CX21), and then the image was reconstructed using the drawing tool of the Power Point software.

## Results

The following results only include those experiments recorded in optimal conditions: pressure, 80–120 mmHg; body temperature, 37°C; end-tidal CO_2_, 3–3.5%; constant urinary output along the experiment; and no evidence of edema or ischemia in the cerebellum. First, we recorded the neuronal population activity on the surface of the cerebellar hemispheres and the vermis to examine whether these regions exhibited electrical sinusoidal potentials, as observed previously in the lumbar spinal cord during fictive scratching [1]. Second, we analyzed the sinusoidal cerebellar activity and its relationship to the activity of the CPG for scratching. Third, we explored the effects of selective sections of the spinal tracts that convey information of the CPG for scratching to the cerebellum.

### Occurrence of electrical sinusoidal activity in the cerebellar cortex during fictive scratching

**Figure 2:**
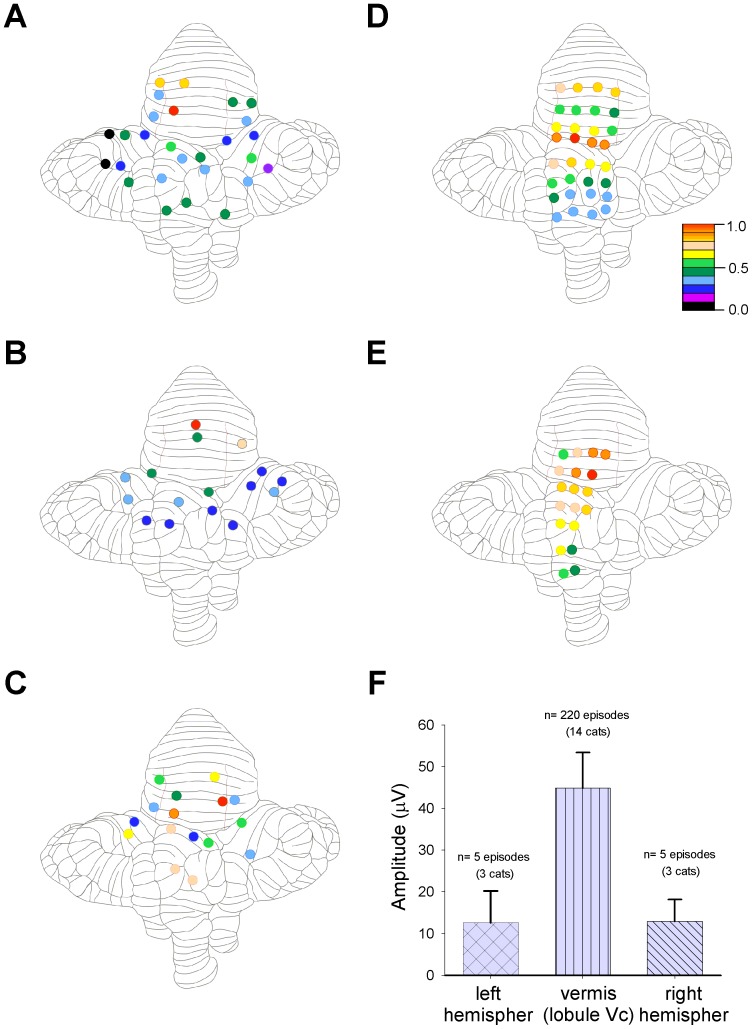
Comparative distribution of the normalized maximum amplitude of the sinusoidal cerebellar potentials (SCPs) during scratching. ***A–E:*** Unfolded cerebellar cortex for 5 cats. The *A–C* maps show the placement of a multi-electrode array on the hemispheres and vermis, and the D–E maps display a multi-electrode array placed on the vermis, for different experiments. The amplitude of the SCPs is denoted by the color code according to the bar in the inset (the highest in red). The amplitude (peak to peak) of the SPCs is measured from averaged (n = 15) recordings from the normalized cycles. The highest amplitude is recorded on the culmen area in all the experiments. ***F:*** summary plot showing the average amplitude of maximal activity recorded in the vermis (lobule Vc; n = 14 cats) and the average amplitude recorded from electrodes placed on the left and right hemispheres, as indicated (n = 3 cats).

The right panel in Figure 1A shows from top to bottom the SCPs (traces 1–27) recorded before and during a scratching episode evoked by the stimulation of the left pinna. The color nomenclature and electrode numbers are depicted in the unfolded cerebellar cortex (left panel). Lower traces in Figure 1A (right panel) show the ENG activity of the extensor MG (blue) and flexor TA (red) nerves during the scratching episode. During the phasic activity of scratching, the SCPs exhibited the largest amplitude in the anterior lobe cortex (dark green and purple traces; 7, 10 and 11), but also smaller amplitudes of the SCPs were recorded on the left and right hemispheres (light green and olive traces; 1–6 and 23–27). The SCPs recorded along the vermis (traces 10–18) were more conspicuous (Figure 1A and 1C) compared to those recorded on the cortices of the paravermis and the hemispheres. Figure 1C compares the amplitude of normalized (based on the flexor cycle phase) and averaged SCPs of 27 recordings along the cerebellar surface from the single episode of scratching shown in Figure 1A (see methods). The region where the SCPs exhibited the highest amplitude was the V lobule (electrode 11). We found a similar distribution of SCPs in four more experiments (Figure 2A–E).

Figure 2A–E illustrates distribution of the normalized maximum amplitude of SCPs on an unfolded cerebellum from five cats. The normalized amplitudes of the SCPs are presented as colored circles according to the scale shown in the inset. Note that the larger amplitude activity occurs on the vermis of the anterior lobe rather than on the hemispheres. The plot in Figure 2F shows the mean amplitude of the SCPs recorded on the culmen area (lobule Vc; 44.8±8.5 µV, n = 5452 cycles averaged from 226 scratching episodes of 14 cats), that was higher than the mean amplitude of those SCPs recorded on the left and right hemispheres (12.6±7.6 µV and 12.85±5.3 µV, respectively). These experiments demonstrate that during a fictive scratching episode there is a population neuronal sinusoidal activity recorded broadly on the surface of the cerebellar vermis and hemispheres.

### Analysis of the electrical SCPs in terms of the CPG for scratching

**Figure 3:**
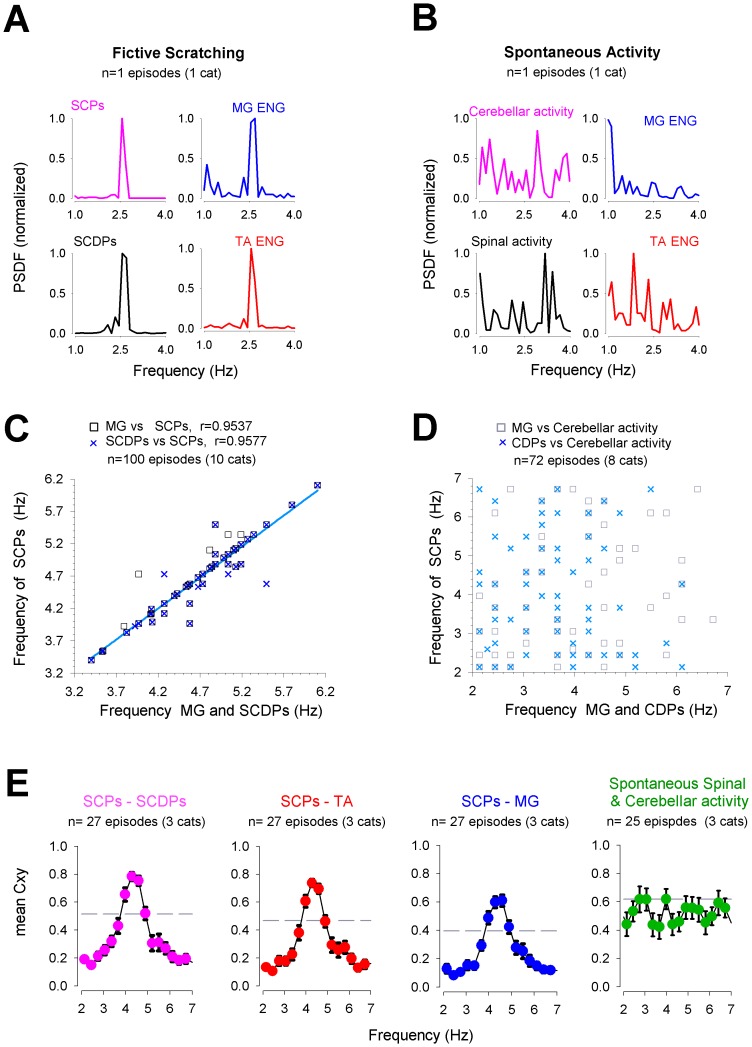
The sinusoidal cerebellar potentials (SCPs) are associated with the activity of the central pattern generator (CPG) for scratching. ***A:*** An example of the power spectral (PSDF) analysis computed for the SCPs, the sinusoidal cord dorsum potentials (SCDPs; arrows heads in *Figure 4A*), and the electroneurograms (ENGs) from the scratching episode shown in *Figure 4A*. Notice the common high pick of power for all the PSDF only at the scratching frequency (2.5 Hz). ***B:*** During spontaneous activity (in the absence of rhythmic activity) the PSDFs computed for the SCPs, SCDPs and ENGs do not exhibit a common high pick of power. The Power density is normalized to the maximal power for each individual graph shown in *A* and *B*. ***C–D:*** Summary plots correlating the dominant frequency (as shown in *A* and *B*) of the cerebellar activity versus the spinal and ENG activity (SCDPs and MG-ENG) during scratching (*C*) and spontaneous activity (*D*) for different animals. Plot *in C* shows a high correlation (r = 0.95, n = 10 cats) between SCPs versus SCDPs and MG-ENG during scratching. In contrast, during spontaneous activity there is no correlation, as shown in *D*. ***E:*** Mean coherence function (Cxy) under the same conditions as panel *A* (n = 3 cats). Note the high coherence between SCPs–SCDPs (pink plot), SCPs-TA (red plot) and SCPs-MG (blue plot) within the scratching frequency range (2–5 Hz). Spontaneous activity does not exhibit an associated common domain frequency (green plot).

**Figure 4:**
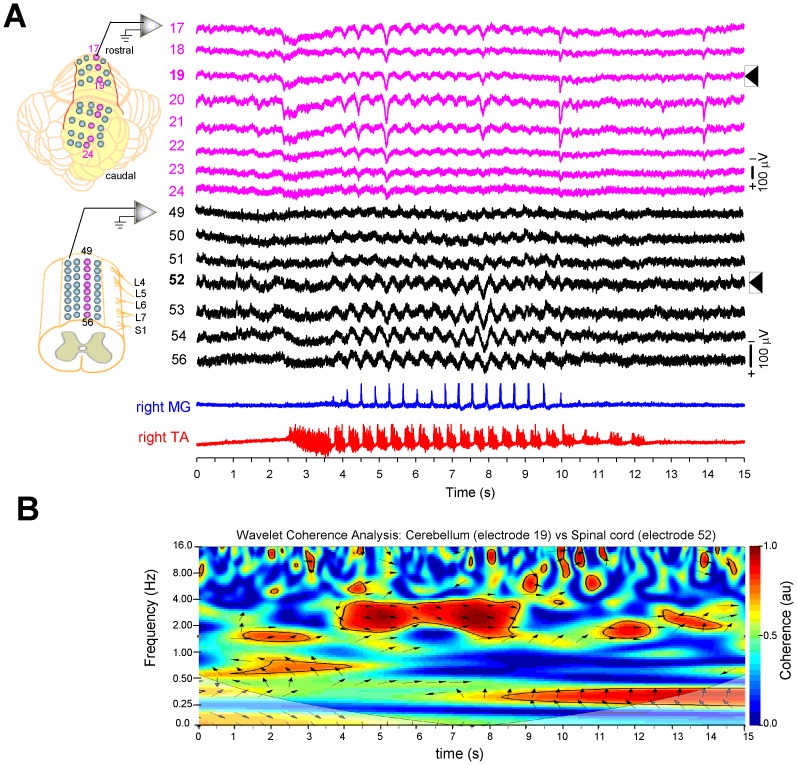
The sinusoidal cerebellar potentials (SCPs) are highly correlated in time and frequency with the sinusoidal cord dorsum potentials (SCDPs). ***A:*** Zoom-out amplification of sixteen recordings from two 32-multielectrode arrays placed on the vermis and the lumbar spinal cord, as shown in the corresponding schematics on the left. Notice that the cerebellar (pink recordings) and the spinal (black recordings) sinusoidal activity co-occur during the scratching episode. ***B:*** Wavelet-transform coherence (WTC) analysis computed between SCPs and SCDPs shown in *A* (arrow heads) reveals a high coherence (red area) around 2.5 Hz only during the rhythmic alternating activity recorded in the ENGs (4–9 s). There is an absence of correlation at the scratching frequency between SCPs and SCDPs during spontaneous activity, or when the ENG rhythmicity is missing. There are some other small spots of coherence, but not related to the sinusoidal activity. Arrows show a phase relationship between both signals, see methods for more details.

We performed three different quantitative analysis techniques to examine whether there is a correlation between the spinal CPG activity and the SCPs. First, we examined the frequency response of the spinal, the cerebellar, and the ENG activity in two conditions; i) during an episode of fictive scratching, and ii) during the spontaneous activity in resting conditions (Figure 3). We computed the PSDF for the scratching episode displayed in Figure 4A. Left plots of Figure 3A show that both the SCPs and the SCDPs exhibit a sharp peak at 2.5 Hz, which is the same frequency of scratching (MG and TA ENGs, right plots). In contrast, both the spontaneous activity recorded in the cerebellum and the spinal cord exhibit no peaks at the scratching frequency (Figure 3B). The plot in Figure 3C shows a high linear correlation (n = 100 scratching episodes from 10 cats) for the SCPs frequency versus the SCDPs frequency (r = 0.96), and for the extensor MG activity versus the SCPs (r = 0.95) across several scratching episodes. In contrast, there is no correlation for the spontaneous cerebellar and spontaneous spinal (CDP) activity as shown in Figure 3D (n = 72 scratching episodes from 8 cats).

A second approach to assess the association between spinal and cerebellar activity was the calculation of the mean coherence (C_xy_) function. Figure 3E shows a high synchronization between the SCPs and SCDPs at 4.3 Hz (mean coherence 0.78, ±0.15, mean ± sd, pink dots), between the SCPs and the flexor, TA (mean coherence 0.74, ±0.14, red dots), and between the SCPs and the extensor, MG (mean coherence 0.61, ±0.20, blue dots) ENGs during fictive scratching (n = 27 scratching episodes, three cats). In contrast, there is no conspicuous coherence between the spinal and the cerebellar spontaneous activity in resting conditions at any frequency around the scratching range (n = 25 episodes, 3 cats; green dots).

Finally, we quantified the spino-cerebellar correlation between the SCDPs and SCPs by means of the time-frequency analysis achieved by the WCT. This analysis allows us to determine the spino-cerebellar coherence between rhythmic patterns in terms of the frequency content in our signals in a given time series [27], [31]. In order to compute the spino-cerebellar WCT shown in Figure 4B, we compared the highest amplitude activity from cerebellum and spinal cord recorded in electrodes 19 and 52, placed on the culmen area of the cerebellum and on the L5–L6 spinal border, respectively (Figure 4A; arrow heads indicate such recordings). In Figure 4B, the X-axis represents the time course of the recording, the same as shown in Figure 4A; the Y-axis represents the frequency domain, for red areas corresponding to the highest coherence correlation. The black arrows indicate the phase correlation (see methods). We found that the predominant high coherence occurs at the scratching frequency band (around 2.5 Hz in this example) over the time course of the episode, from 4 to 9 seconds, where the SCPs also exhibited the highest amplitude. Nonetheless, before and after the scratching episode, 0–4 sec and 9–15 sec, we found some spots of coherence but with frequencies below or above 2.5 Hz. The same profile of this time-frequency correlation was also evident in four more experiments analyzed. However, as the dominant frequency for scratching varies among different episodes, it is not possible to construct a summary wavelet plot with this type of analysis.

Therefore, we conclude that during the rhythmic motor task of scratching there is a spino-cerebellar synchrony between the SCDPs and SCPs, preferentially, at the scratching frequency. These data indicate that there is a neuronal correlate of the spinal CPG for scratching in the cerebellum that we recorded as a SCP.

### Selective sections of spinocerebellar tracts that convey central pattern generator information during scratching

**Figure 5:**
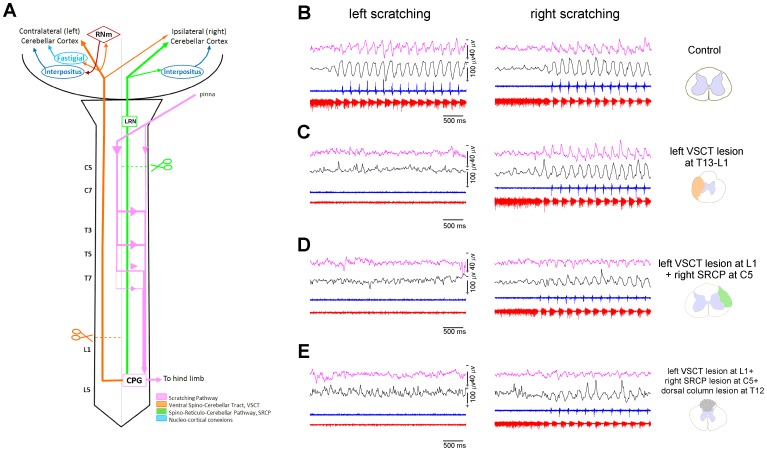
Disruption of spino-cerebellar pathways alters the projection of the scratching CPG information to the cerebellum. ***A:*** The diagram shows the scratching pathway (pink line), the spino-cerebellar pathways that convey the CPG information to the cerebellar cortex (ventral spinocerebellar tract, VSCT, orange; spinoreticular cerebellar pathway, SRCP, green) and the spinal levels where the sections were performed (scissors). ***B–E:*** From top to bottom: cerebellar sinusoidal potentials (SCPs, pink), sinusoidal cord dorsum potentials (SCDPs, black), extensor (blue) and flexor (red) electroneurograms. *B:* Left and right panels show the control episodes of left and right scratching, respectively. ***C:*** After sectioning the left VSCT, the left scratching disappears because of the disruption of the left scratching pathway, while right scratching remains undisturbed. ***D:*** After sectioning both the VSCT and the SRCP the SCPs persist; although they exhibit lower amplitude than during control scratching. ***E:*** The additional section of the dorsal columns has no influence on the remaining SCPs because there is no projection to the cerebellum through dorsal column tracts.

As mentioned in the Introduction, the VSCT and the SRCP tracts convey information from the spinal CPG to the cerebellum. The VSCT and SRCP information ascends in the ventral gray matter of the lateral funiculus, contra- and ipsi-laterally, respectively [9], [10], [11], [30], see Figure 5A. The anatomical characteristic of these pathways allows us to section them selectively without eliminating the scratching in the stimulated side, thus disclosing the contribution of each pathway in the generation of SCPs. The pink line, in the scheme of Figure 5A, illustrates the scratching pathway described by Delyagina and colleagues [32]. Such a pathway runs preponderantly contralateral to the stimulated pina to evoke scratching, then it crosses back again to the ipsilateral side at the spinal thoracic level (T3–T7), reaching the lumbosacral spinal CPG circuits, and finally, producing scratching in the ipsilateral stimulated side (Figure 5B; control); only a few axons run along the ipsilateral side.

**Figure 6:**
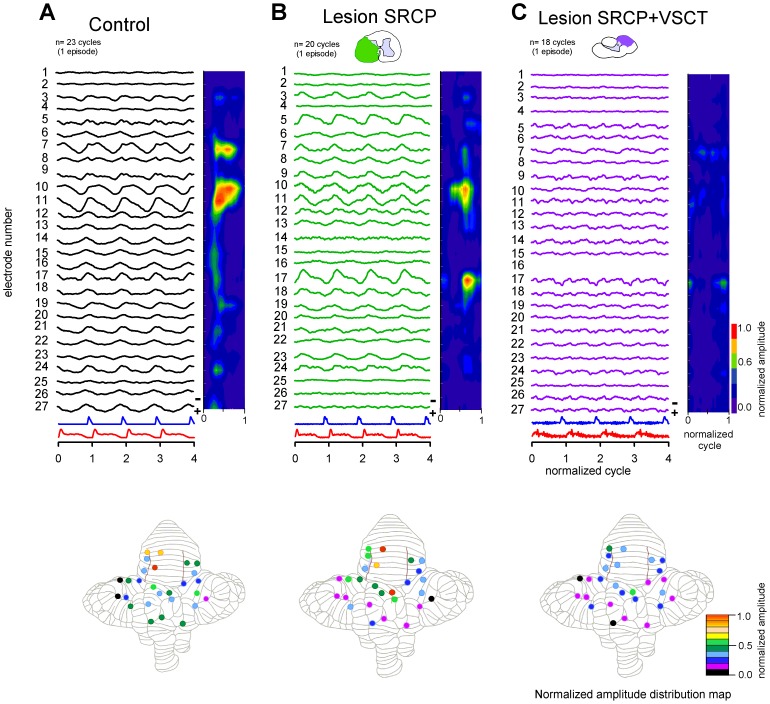
The sinusoidal cerebellar potentials (SCPs) persist after disruption of the ventral spinocerebellar tract (VSCT) and the spinoreticular cerebellar pathway (SRCP). ***A–C:*** Averaged and normalized SCPs (traces) recorded with the multielectrode array, as shown in *Figure 1A*. For clarity, the averaged and normalized scratching cycle is displayed four times. The contour plots on the right show the amplitude from left traces, which was normalized to the highest control amplitude (trace 11 in *A*). In addition, the lower maps for each panel show the distribution of maximal normalized amplitude for the *A–C* traces. ***A:*** *Black traces*, control SCPs during one episode of scratching. The SCPs with the highest amplitude (traces 10–11 and red color spot on the right plot) were recorded on the culmen lobule. ***B:*** *Green traces*, SCPs after section of the SRCP. The extent of the lesion at C5 spinal segment is illustrated in the histological reconstruction (green area) on the top. Notice that under this condition the SCPs with the highest amplitude were recorded on the culmen (traces 10–11), but also on the lobule VI (trace 17). ***C:*** *Purple traces*, SCPs after section of both the SRCP and VSCT (purple area on the top histological reconstruction). The sinusoidal activity persists on the vermis (traces 10–11 and trace 17) although with a notorious amplitude decrease. Missing trace 16 was not recorded.

**Figure 7:**
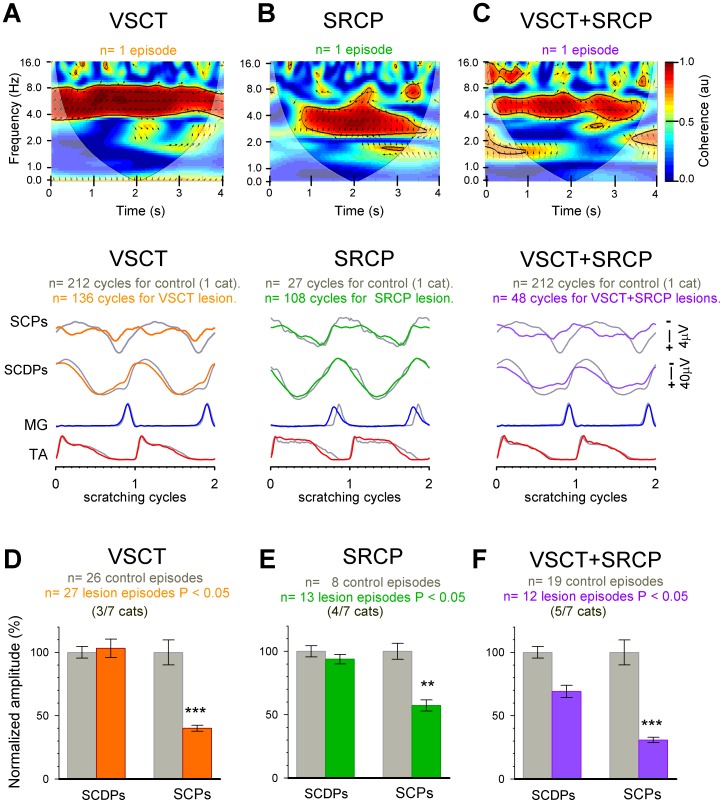
After section of the ventral spinocerebellar tract (VSCT) and the spinoreticular cerebellar pathway (SRCP), the sinusoidal cerebellar potentials (SCPs) are still correlated with the spinal CPG activity. ***A–C:*** *Upper panels*, wavelet-transform coherence (WTC) analysis computed between SCPs and SCDPs after sectioning the VSCT (*A*), the SRCP (*B*) and both VSCT+SRCP (*C*). *A* and *C* are from the same experiment, and *B* from a different experiment. Under all conditions there is a spino-cerebellar correlation in time and frequency at the scratching frequency (∼4 Hz). *Lower panels*, from top to bottom, SCPs, SCDPs, and MG and TA ENGs under the same conditions (solid lines) and before sections (control, gray lines). All traces were averaged and normalized based on the flexor (TA) cycle of scratching. ENGs were also rectified. For clarity, two scratching cycles are displayed. The control traces are superimposed to show the amplitude decrease after lesions. Note that the SCPs are reduced after individual sections of the tracts. ***C–F:*** Summary plots of the effects after sectioning the VSCT, SRCP and both VSCT+SCRP for different experiments. Average amplitude for control (gray bars) versus VSCT (*D*; orange bars, n =  3/7 cats), SRCP (*E*; green bars, n = 4/7 cats) and VSCT+SRCP (*F*; purple bars, n = 5/7 cats) sections. The amplitude of the SCPs decreases significantly (P<0.05; Mann-Whitney U test) after the three sections (VSCT by 42.7±6.3%; SRCP by 60±10%; and VSCT+SRCP by 69.1±10). The amplitude of the SCDPs is only reduced (30.8±5.6%) after VSCT+SRCP lesion, with no significant change for individual sections. Statistically significant differences in amplitude reduction among the VSCT, the SRCP and VSCT+SRCP groups are indicated by the asterisks (***, P<0.001; **, P<0.01) as calculated using the Kruskal-Wallis test followed by Dunn's test.

Therefore, section of the lateral funiculus, contralateral to the scratching side, at T13-L1 spinal cord level (orange scissors, in Figure 5A), leads to disruption of the VSCT (orange line in Figure 5A). We observed that the SCPs recorded in the cerebellar cortex persist throughout the scratching episode (Figures 5C, right panel). As expected, the contralateral scratching was eliminated (Figures 5C, left panel). Similar results occurred when we transected the SRCP at the ipsilateral C5 spinal level (green scissors in Figure 5A) and the VSCT remained intact (see Figure 6B). However, even when both the VSCT and SRCP were transected, the sinusoidal cerebellar activity remained during scratching with lower amplitude (Figure 5D; Figure 6C; Figure 7C and G). We observed no additional changes in the scratching-evoked SCPs and SCDPs after sectioning the dorsal columns (Figure 5E).

In the experiment shown in Figure 6, we sectioned first the SRCP and then the VSCT. Figure 6A shows the averaged SCPs (based in the normalized cycle) for one episode of scratching in control conditions. Figures 6B and C show the effects of transections of the SRCP, and SRCP plus VSCT, respectively. At the top of each panel in Figure 6B and C we show schematics with the extent of spinal sections. We excluded from the analysis those recordings in which the signal to noise ratio decreased after performing the sections. After sectioning the SRCP, the rhythmic sinusoidal cerebellar activity persisted, but exhibited smaller amplitudes (Figure 6B, green traces). The additional section of the VSCT reduced even more the amplitude of the SCPs (Figure 6C, purple traces), but still displaying rhythmicity coupled to the ENG activity. Additionally, we plotted the contour graphs to show the amplitude distribution of the SCPs along the cerebellar cortex before and after sectioning the SRCP, and both the SRCP plus VSCT (Figure 6A–C, right contour plots). We observed a change in the distribution of spots with the highest SCPs amplitude after sectioning one, or both tracts (see discussion).

In order to examine if the remaining SCPs recorded after the aforementioned lesions still exhibit synchrony with the spinal CPG activity, we performed a WCT analysis of the cerebellar and spinal recordings (Figure 7) after sectioning: i) the VSCT (Figure 7A), ii) the SRCP (Figure 7B), and iii) both spinocerebellar pathways (Figure 7C). Under these conditions, we observed a spino-cerebellar correlation in the time and frequency domains at the scratching frequency. However, after sectioning both pathways (Figure 7C) there were some spots of high spino-cerebellar correlation at frequencies different from those of the scratching frequency. This can be explained in terms of the disruption of the spinocerebellar loop that controls the scratching coordination (see discussion).

Traces in Figure 7 (lower panels) show from one experiment, the average amplitude of the SCPs and SCDPs, after sectioning the VSCT (orange traces; n = 136 cycles), the SRCP (green traces; n = 108 cycles), and both tracts (purple traces; n = 48 cycles), compared to the respective controls (gray traces). We found a significant reduction in amplitude of the SCPs after individual sections of the VSCT, the SRCP, and both tracts. The amplitude of the SCDPs was reduced only after sectioning both tracts (Figure 7C, middle panel). We obtained similar results across all the experiments, as summarized in Figure 7, where the VSCT was sectioned first in 3/7 (Figure 7D), SCRP was sectioned first in 4/7 (Figure 7E), and both VSCT and SRCP in 5/7 experiments (Figure 7F). The averaged responses reveal a significant (Mann–Whitney U test; P<0.05) decrease in amplitude of the SCPs with respect to the controls (VSCT by 42.7±6.3%; SRCP by 60±10%; and VSCT+SRCP by 69.1±10%). The amplitude of the SCDPs showed no significant differences for individual sections, but it was reduced significantly (by 30.8±5.6%) when both tracts were sectioned (Figure 7F).

We compared the effects of sectioning the VSCT and the SRCP on the SCPs amplitude by using the Kruskal-Wallis test (***, P<0.001; **, P<0.01), and we found no significant differences (Figures 7D and F). Therefore, it seems that both the VSCT and the SRCP contribute similarly to the activation of neuronal populations in the cerebellar cortex, that finally generate the sinusoidal activity (see discussion).

## Discussion

We found that during scratching, the sinusoidal electrical potentials (SCDPs) recorded on the lumbar spinal cord occurs in synchrony with the sinusoidal cerebellar potentials (SCPs) recorded on the surface of the cerebellar cortex. The section of the SRCP and the VSCT did not abolish the amplitude of the SCPs, suggesting that there are additional pathways conveying information from the spinal CPG to the cerebellum.

Based on the studies reporting a rhythmical modulation of Purkinje cells recorded in the hindlimb projection zone of the cerebellar cortex [14], [15], [16], [18], [19], we expected that the SCPs were restricted to the same zone. However, we recorded the SCPs beyond the described hindlimb and spinocerebellar projecting areas [5], [6], [7], [33], [34], suggesting that the CPG projections for scratching have a broader representation in the cerebellar cortex than described before (Figure 1 and 2). Since we analyzed the global neuronal cerebellar activity, previously unexplored, our study extends previous electrophysiological reports based on intracellular and unitary activity recordings of Purkinje cells during locomotion and scratching [14], [15], [16], [17], [18], [19], [20], [21].

### Sinusoidal Cerebellar Potentials are generated by the scratching CPG activity

We suggest that the SCPs represent the cerebellar population neuronal activity triggered by the efferent copy of the rhythmic motor activity generated by the spinal CPG for scratching. The first evidence that correlates the SCPs to the CPG activity was revealed by the high correlation of the power spectral density and the significant coherence between spinocerebellar activity, only during the rhythmic component of scratching episodes (alternation of flexor and extensor ENG activity) and not during the tonic phase of scratching or resting conditions. However, to quantitatively assess this assumption, we used correlational methods as indirect indicators of neuronal connections [31], [35], [36]. We found a significant frequency modulation and a rhythmic coupling between the cerebellar and the spinal sinusoidal activity over the whole time course of the scratching episode, and as soon as it finished, the sinusoidal activity subsided.

In order to discard any potential influence on the rhythmic synchrony between the background spinal and cerebellar activity, we analyzed the spontaneous activity before and after scratching and found no correlation, at least around the frequency ranges for scratching. The spino-cerebellar WCT analysis revealed some spots of high correlation not associated with the spinal CPG, since they were neither in the scratching frequency range nor within the scratching episode. Even though we observed very sporadic spontaneous potentials recorded simultaneously on the spinal cord and the cerebellum, but they occurred at very low frequencies (unpublished data from our lab). It is known that the cerebellum behaves as a follower of the spinal CGP activity. The possibility that the cerebellum commands the spinal cord to generate the scratching, and the associated spinal sinusoidal SCDPs, is excluded based on classical observations that scratching can be elicited even after the whole transection of the spinal cord at upper cervical levels, or after removing the cerebellum [37], [38], [39], [40], [41], [42], [43].

We propose the SCPs as a measurable parameter of the global cerebellar activity in response to the synaptic driving from the spinal CPG. Such SCPs could be useful for studying the role of the cerebellum in the coordination of motor control during rhythmic tasks. For instance, our results would complement those studies on the cerebellar rhythmic modulation based on unitary and intracellular recordings of Purkinje cells during locomotion [14], [15], [16], [17], [18], [19], [21]. These would also be important to study the spatiotemporal patterns [for review: 42], [43] during coordinated movements after removing the sensory and proprioceptive feedback, in contrast to awake animals [44].

### Distribution of projections from the CPG for scratching to the cerebellar cortex

The wide distribution of the SCPs we recorded on the cerebellar cortex, unveils the possibility that mossy fibers convey information from the spinal CPG to extended areas in the cerebellum. Our results (Figures 5–7) suggest that the spinal neurons producing SCDPs project towards the cerebellum *via* the VSCT, the SRCP, and other possible pathways. Such extensive distribution of these pathways in the cerebellar vermis and paravermis finally produces the sinusoidal SCPs. It means that the CPG for scratching has its own representation in the cerebellum, possibly different to the representation of sensory information from the hindlimbs. This is consistent with previous studies by Arshavsky and Orlovsky's group, who demonstrated experimentally that the VSCT [8], [10], [11], [45], [46] and the SRCP [9], [11] send information to the cerebellum about central events rather than peripheral. They also demonstrated that both pathways are rhythmically modulated during real or fictive locomotion, and scratching.

Early studies, by means of antidromic stimulation and unitary recordings, described that the cerebellar projections of the VSCT [5], [47] mainly terminate contralaterally to their spinal origin. The projection areas involve longitudinal zones in the intermediate cortex, and the lateral region of the vermal cortex, of the anterior lobe. A few fibers also terminate in the paramedian and pyramis lobules, all of them in the same areas as the classical hindlimb areas described by Adrian [33]. Furthermore, fibers from the major portion of LRN project bilaterally in the anterior lobe and pyramis, and ipsilaterally in the paramedian lobule [6], . We found SCPs occurring beyond the aforementioned projections areas (see Figures 1 and 2), mainly on the vermis of the anterior lobe (culmen lobule, V), the vermis in the posterior lobe (folium and tuber lobules, VII), and the cerebellar hemispheres (lobule simplex, H-IV, H-V, and ansiform lobule, H-VII). Some areas showed only a slight sinusoidal activity including the vermis of the anterior lobe (culmen lobule, IV), the vermis of the posterior lobe (declive lobule, VI; pyramis lobule, VIII) and the hemispheres (paramedian lobule, H-VII).

The distribution of SCPs on the cortex does not correspond to the same areas of the VSCT [5]; however, such distribution is consistent with some axon projections areas of the SRCP [6], [7]. Nevertheless, because the SCPs could result from the impinging activity of the CPG for scratching, that projects ipsi- and contralaterally to the cerebellum, our findings partially agree with such previous reports about the projections of the SRCP and VSCT.

Although in our experiments we recorded the SCPs on the lobules; VII, H-IV and H-V, where neither the VSCT nor the SRCP projections have been reported before, there are some studies that support our findings [15]. The distribution of the SCPs that we found in the present study is in agreement with the widespread distribution of cerebellar cells modulated by stepping movements of the hindlimb, recorded in decerebrate animals during passive movements [49], [50], or animals walking on a treadmill [51]. Furthermore, immunohistochemical studies in our lab (unpublished data) [52] and those obtained by Jasmin and colleagues [53] are also in agreement with our findings. In addition, these authors also reported densely labelled cerebellar areas for c-Fos expression in awake walking rats on a rota-rod. However, some caution must be taken into account because the sensory and proprioceptive feedback conveyed by the Dorsal Spino-Cerebellar Tract (DSCT) was kept intact, whose projections overlap with the VSCT and the SRCP areas. Therefore, we also considered the possibility that SCPs represent the global cerebellar activity in response not only to VSCT and SRCP, but also to additional spinocerebellar pathways involved in the transmission of the CPG information.

### The sinusoidal cerebellar activity persists after selective sections of the VSCT and the SRCP

The significant decrease in the amplitude of the SCPs, after separate transections of the VSCT and the SRCP, suggests a reduction of the CPG's synaptic driving from the spinal cord to the cerebellum, but not of the CPG activity itself because there was no significant decrease the amplitude of the SCDPs. We observed that the decrease in amplitude, due to VSCT disruption, was not significantly different from the decrease due to the SRCP disruption, as found by Arshavsky and colleagues [11]. However, in addition to those findings [9], [10], [11], we suggest that there are additional pathways that convey information from the scratching CPG, based on the persistence of SCPs recorded after transecting both the VSCT and the SRCP. Thus, the question that arises in regard to the pathways that convey the spinal CPG information to the cerebellum besides the VSCT and the SRCP. Plausible spinocerebellar pathways involved could be the DSCT, the Spino-Olivocerebellar Pathways (SOCP) and the Spino-Rubral Pathway (SRP).

Recently, Stecina and colleagues [54] showed that the DSCT provides the cerebellum not only with proprioceptive sensory information, but also with rhythmic central inputs from the CPG. However, we have to remark that we also disrupted the DSCT when we sectioned the VSCT, as well as the dorsolateral funiculus, which conveys axons from the DSCT. Therefore, we suggest the DSCT is not the source of the remaining sinusoidal activity we observed. Another possible source of the spinocerebellar pathway that conveys information from the CPG activity is the SOCP, as suggested by Smith [55] when recording olivary neurons that discharged rhythmically during treadmill locomotion. Nevertheless, Armstrong [20], Orlovsky [30], and Udo [17] established that the role of SOCP is to transmit sensory information of stepping-related events. This pathway runs bilaterally and diffusely in the ventral tract, therefore it is not possible to be sectioned selectively, and more specifically, in our experiments it is not possible to cut the SOCP without disrupting the scratching pathway, given that this pathway remains as a candidate for transmission of CPG information. Finally, it is known that the SRP, a spinocerebellar pathway, not well studied, runs ipsilaterally through the ventral funiculus to reach the magnocellular red nucleus (RNm), and then to the interpositus cerebellar nucleus to conform a cerebellar nuclei-cortex loop that informs about both somatosensory and CPG activity related to locomotion [56]. Thus, this seems to be a second candidate for the remnant sinusoidal activity, and, as in the case of the SOCP, we could not disrupt this pathway selectively without damage of the scratching pathway.

### Physiological implications

Albeit projections of sensory fibers from hindlimbs partially share a common distribution to the pathways transmitting CPG activity to the cerebellum [6], [7], [9], [10], our results highlight a broader distribution of CPG projections to the cerebellum. These findings reveal that more areas are involved in the integration of a motor program (i.e. scratching and locomotion) for processing proprioceptive information and motor programs, in order to compare and adjust the precision of movements according to the environmental demands [for review of cerebellum role in motor coordination see 4].
